# Salvianolic acid C potently inhibits SARS-CoV-2 infection by blocking the formation of six-helix bundle core of spike protein

**DOI:** 10.1038/s41392-020-00325-1

**Published:** 2020-10-06

**Authors:** Chan Yang, Xiaoyan Pan, Xinfeng Xu, Chen Cheng, Yuan Huang, Lin Li, Shibo Jiang, Wei Xu, Gengfu Xiao, Shuwen Liu

**Affiliations:** 1 Guangdong Provincial Key Laboratory of New Drug Screening, School of Pharmaceutical Sciences, Southern Medical University, Guangzhou, China; 2 State Key Laboratory of Virology, Wuhan Institute of Virology, Center for Biosafety Mega-Science, Chinese Academy of Sciences, Wuhan, China; 3 Key Laboratory of Medical Molecular Virology (MOE/NHC/CAMS), School of Basic Medical Sciences, Fudan-Jinbo Joint Research Center, Fudan University, Shanghai, China; 4 State Key Laboratory of Organ Failure Research, Guangdong Provincial Institute of Nephrology, Southern Medical University, Guangzhou, China

**Keywords:** Drug screening, Target identification

**Dear Editor,**

The pandemic of COVID-19 caused by SARS-CoV-2 infection has posed a serious threat to global public health and the economy. Up to now, although several potentially effective antiviral drugs are under evaluating in clinical trials around the world,^1^ there are still no specific antiviral countermeasures beyond supportive therapies have been established. We herein report that the hydrophilic compound Salvianolic acid C (Sal-C) from Danshen, a traditional Chinese medicine (TCM), potently inhibit SARS-CoV-2 infection by blocking the formation of six-helix bundle (6-HB) core of spike (S) protein.

The spike protein of SARS-CoV-2 plays a key role in receptor recognition and virus-cell membrane fusion and shows a great efficiency in mediating virus entry, which is consisted of S1 and S2 subunits. After binding to the cell receptor via receptor-binding domain (RBD) in S1, SARS-CoV-2 S2 will change its conformation by forming a 6-HB between HR1 and HR2 (two main components of S2 subunits) domains, leading to viral membrane fusion.^2^ In view of the high transmission rate and infection rate of SARS-CoV-2, we focused on the S2 subunit with highly conservative properties as a target to develop small-molecule inhibitors for SARS-CoV-2 S-mediated cell–cell fusion.

Based on our previous studies on seeking for h-CoVs fusion inhibitors,^3,4^ we utilized the cell–cell fusion assay mediated by SARS-CoV-2 S protein to screen the TCM monomer library for discovering fusion inhibitors. And, Sal-C was identified to potently inhibit the membrane fusion of S-overexpressed-HEK293T and Vero-E6 cells with half maximal inhibitory concentration (IC_50_) of 1.71 μM (Fig. 1a, b and Supplementary Fig. S1a).

**Fig. 1:**
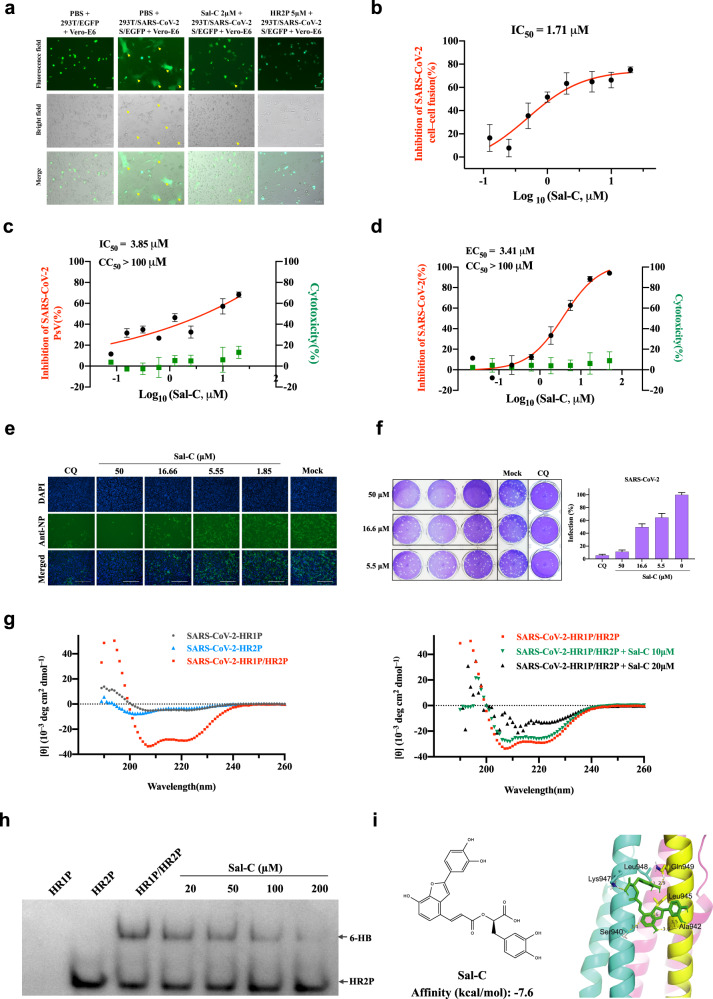
Sal-C inhibits SARS-CoV-2 infection by blocking the formation of six-helix bundle core of S protein. **a** SARS-CoV-2 S protein-mediated cell–cell fusion assay. The syncytium of Vero-E6 cells and HEK293T cells with SARS-CoV-2 S overexpression were marked in the pictures. HR2P was used as a positive control. Representative results were shown from three fields were selected randomly each sample with scale bars of 100μm. **b** Inhibitory activities of Sal-C on SARS-CoV-2 S protein-mediated cell–cell fusion. **c** Sal-C inhibited the entry of SARS-CoV-2 S PsV on 293T/ACE2 cells. **d** Validation on the antiviral activity of Sal-C against authentic SARS-CoV-2 in Vero-E6 cells. The inhibitory curve and cytotoxic effect for Sal-C. **e** The inhibition activity of Sal-C on SARS-CoV-2 infection (green) was detected by indirect immunofluorescence assay. The nuclei (blue) were stained with DAPI, scale bar=200μm. **f** Plaque reduction assay of Sal-C against authentic SARS-CoV-2 in the Ongoing-infection model. **g** Sal-C inhibited α-helical conformation change. CD spectra of SARS-CoV-2 HR1P alone (gray), SARS-CoV-2 HR2P alone (blue), and SARS-CoV-2 HR1P/HR2P complex (red) in phosphate buffer (pH 7.2) at 4°C. The typical α-helicity conformation was significantly interfered with Sal-C, as shown in the black and green model, with minimum values at 208 or 222nm. **h** Determination of the 6-HB between SARS-CoV-2 HR1P and HR2P by 18% N-PAGE. HR1P (25µM) with or without Sal-C were incubated at 25°C for 30min, followed by the addition of HR2P (25µM). The mixture was incubated at 25°C for another 30min before being loaded into the gel. Sal-C reduces the density of the 6-HB. **i** Chemical structure and schematic diagram of molecular docking between Sal-C and the post-fusion core of 6-HB. The affinity of Sal-C with the post-fusion core of 6-HB was −7.6kcal/mol

Pseudovirus (PsV) system is a classic model to study the entry process of envelop viruses, as well as to assess the activity of antiviral agents targeting the virus entry stage. Here, we developed a PsV system using SARS-CoV-2 S protein to study the virus entry (Supplementary Fig. S2a) and tested Sal-C on this assay. As a result, Sal-C was determined to inhibit the entry of SARS-CoV-2 PsV with IC_50_ of 3.85 μM on HEK293T cells stably expressing human-ACE2 (Fig. 1c). The results on Vero-E6 cells (Supplementary Fig. S2b) were in accordance with that on HEK293T cells, while no inhibitory activities were observed on vesicular stomatitis virus glycoprotein (VSV-G) PsV under the treatment of Sal-C (Supplementary Fig. S2c) and Chloroquine (CQ) was used as the positive drug control (Supplementary Fig. S2d).

To confirm the inhibitory effects of Sal-C on SARS-CoV-2, we performed authentic SARS-CoV-2 inhibition assays in a BSL-3 facility. We determined EC_50_ of Sal-C against authentic SARS-CoV-2 on Vero- E6 cells with a full-time treatment model. As shown in Fig. 1d, Sal-C showed the potent antiviral activity with EC_50_ of 3.41 μM. Consistently, Sal-C inhibited SARS-CoV-2 infection in a dose-dependent manner as observed in Fig. 1e, which were detected by indirect immunofluorescence assay against SARR-CoV-2 N protein. Furthermore, we found that Sal-C significantly reduced the number of plaques in the Ongoing-infection model (Fig. 1f) but not in the Post-infection model (Supplementary Fig. S2e), confirming that Sal-C inhibits SARS-CoV-2 infection by targeting the viral entry stage.

The formation of the 6-HB fusion core is a key step in SARS-CoV-2 S-mediated membrane fusion. Peptides derived from HR2 regions of SARS-CoV-2 are the earlier fusion inhibitors as reported.^5^ These previous studies raise confidence about whether Sal-C as a potential SARS-CoV-2 fusion inhibitor targeting the highly conserved HR1 or HR2 region. Subsequently, HR1P and HR2P, two peptides overlapping the interacting regions of HR1 and HR2 fusion core (Supplementary Fig. S2f–g), were synthesized to identify the anti-SARS-CoV-2 mechanism of Sal-C. We determined the biophysical change of 6-HB by using circular-dichroism (CD) spectroscopy and native-polyacrylamide gel electrophoresis (N-PAGE) analysis as described before.^5^ While the SARS-CoV-2 HR1P/HR2P complex exhibited the typical α-helicity of 6-HB, HR1P alone or HR2P alone exhibited low helicity, and the characteristic α-helicity of 6-HB was disrupted with the treatments of Sal-C dose-dependently (Fig. 1g). On the other hand, as shown in Fig. 1h, HR2P peptide alone showed a clear band at the lower position. When HR2P was mixed with HR1P, a specific and visible band at the upper position corresponding to the 6-HB structures was revealed on the gel. The density of the 6-HB (upper bands) decreased with increasing concentration of Sal-C, while the density of the unbound HR2P (lower bands) increased. These results give evidence that Sal-C inhibits the infection of SARS-CoV-2 by disturbing the formation of 6-HB between HR1P and HR2P.

To identify the possible binding sites for Sal-C, we docked Sal-C into the 6-HB domain. In the docked structures, the binding affinity for Sal-C was 7.6 kcal/mol (Fig. 1i). For binding details in the docked structure, Sal-C can interact with residues Ser940, Thr941, Ala942, Leu945, Lys947, Leu948, and Gln949 in the HR1 pocket of the 6-HB core, providing insight into its molecular structure relationship with the 6-HB core region. Consistent with the docking results, Sal-C showed no effect on inhibition of SARS-CoV-2 S binding to the ACE2 receptor (Supplementary Fig. S3a). Additionally, we determined the binding affinities between Sal-C and S, S2 or RBD protein. The result showed that Sal-C bound to SARS-CoV-2 S and SARS-CoV-2 S2 proteins with similar binding affinity (at micromolar level), while the binding between SARS-CoV-2 RBD and Sal-C showed much lower binding affinity (at millimolar level, Supplementary Fig. S3b–d). These data suggested that Sal-C has the tendency to bind to the region (s) in S protein S2 subunit that participate in the 6-HB formation.

Collectively, Sal-C, as a potential small-molecular fusion inhibitor, inhibits SARS-CoV-2 infection by binding to the conserved hydrophobic pocket in the SARS-CoV-2 HR1 region at the fusion-intermediate state and blocking 6-HB formation between HR1 and HR2. As the anti-inflammation effects and biological mechanisms of Sal-C have been reported, Sal-C might have a potential effect on the inhibition of cytokine storm induced by SARS-CoV-2, which also needed to be validated in vivo. This study puts forward a potential use of Sal-C for COVID-19 therapies or prophylaxis and provides a basis to design fusion inhibitors against SARS-CoV-2 infection.

## Supplementary information

Supplemental Materials
