# Supplementary material for: Salvianolic acid C potently inhibits SARS-CoV-2 infection by blocking the formation of six-helix bundle core of spike protein
Source: Signal Transduct Target Ther. 2020 Oct 6;5:220. doi: 10.1038/s41392-020-00325-1 (PMC7538051; doi:10.1038/s41392-020-00325-1)
Supplement: Supplementary file 1 — Supplemental Materials [file 41392_2020_325_MOESM1_ESM.pdf]

Supplemental Materials

Salvianolic acid C potently inhibits SARS-CoV-2 infection by blocking formation of six-helix bundle core of spike protein

Chan Yang[1#], Xiaoyan Pan[2#], Xinfeng Xu[1], Chen Cheng[1], Yuan Huang[1], Lin Li[1], Shibo Jiang[3], Wei Xu[1], Gengfu Xiao[2], Shuwen Liu[1,4]

[1]Guangdong Provincial Key Laboratory of New Drug Screening, School of Pharmaceutical Sciences, Southern Medical University, Guangzhou, China; [2]State Key Laboratory of Virology, Wuhan Institute of Virology, Center for Biosafety Mega-Science, Chinese Academy of Sciences, Wuhan, China; [3]Key Laboratory of Medical Molecular Virology (MOE/NHC/CAMS), School of Basic Medical Sciences, Fudan-Jinbo Joint Research Center, Fudan University, Shanghai, China; [4]State Key Laboratory of Organ Failure Research, Guangdong Provincial Institute of Nephrology, Southern Medical University, Guangzhou, China.

[#]These authors contributed equally: Chan Yang, Xiaoyan Pan

Correspondence:

Shuwen Liu (liusw@smu.edu.cn); Gengfu Xiao (xiaogf@wh.iov.cn); Wei Xu (xuwei3322@smu.edu.cn)

This file includes:

Fig. S1 Effects of Sal-C on inhibition SARS-CoV-2 S-mediated cell-cell fusion

Fig. S2 Miscellaneous for Sal-C inhibiting SARS-CoV-2 PsV and authentic virus infection

Fig. S3 Sal-C interacts with SARS-CoV-2 spike protein S2 subunit

Table. S1 Binding affinities of Sal-C to SARS-CoV-2 S-Fc, SARS-CoV-2 S2-Fc and SARS-CoV-2 RBD-Fc

Materials and Methods

Supplemental References

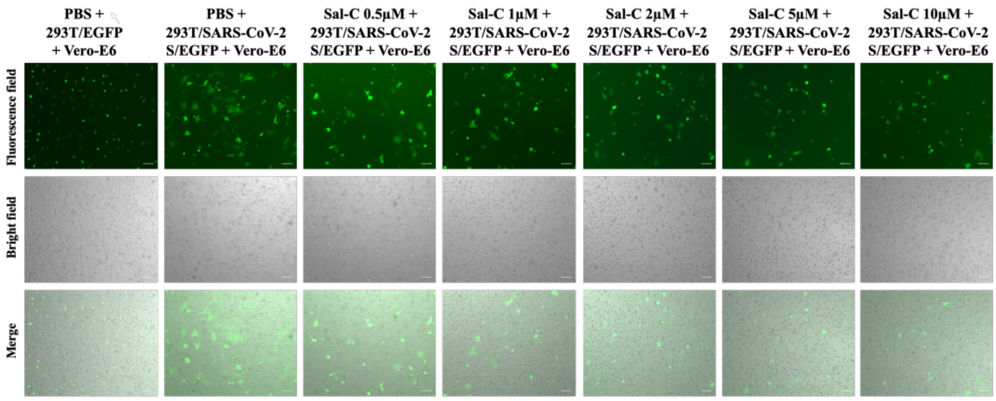

Fig. S1 Effects of Sal-C on inhibition SARS-CoV-2 S-mediated cell-cell fusion. Schematic representation of SARS-CoV-2 S protein-mediated cell-cell fusion. 293T cells were transfected with plasmid pAAV-SARS-CoV-2-S-IRES-EGFP to naturally present the viral S protein on cellular membrane surface and EGFP in cellular cytoplasm (293T/SARS-CoV-2 S/EGFP). Vero-E6 cells were used as target cells. scale bar = 200 μm.

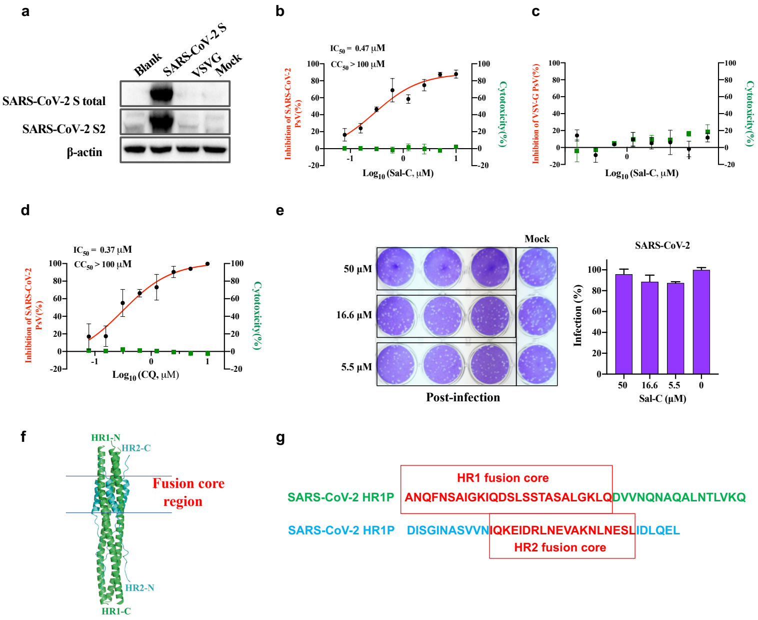

Fig. S2 Miscellaneous for Sal-C inhibiting SARS-CoV-2 PsV and authentic virus. a Detection of SARS-CoV-2 S protein in cells lysate by western blot. Blank, untreated HEK293T cells. Mock, HEK293T cells transfected with empty vector (pNL4-3.Luc.R-E-). The VSV-G PsV were used as a negative control. **b** Sal-C inhibited the entry of SARS-CoV-2 S PsV on Vero-E6 cells. **c** Sal-C showed no effect on the entry of VSV-G PsV. **d** Chloroquine (CQ) inhibited the entry of SARS-CoV-2 S PsV on 293T/ACE2 cells. **e** Plaque reduction assay of Sal-C against authentic SARS-CoV-2 in the Post-infection model. **f** Crystal structure of SARS-CoV-2 6-HB and the fusion core regions are indicated.[1] **g** Amino acid (aa) sequences and the 6-HB core regions of SARS-CoV-2 HR1 and SARS-CoV-2 HR2 peptides.

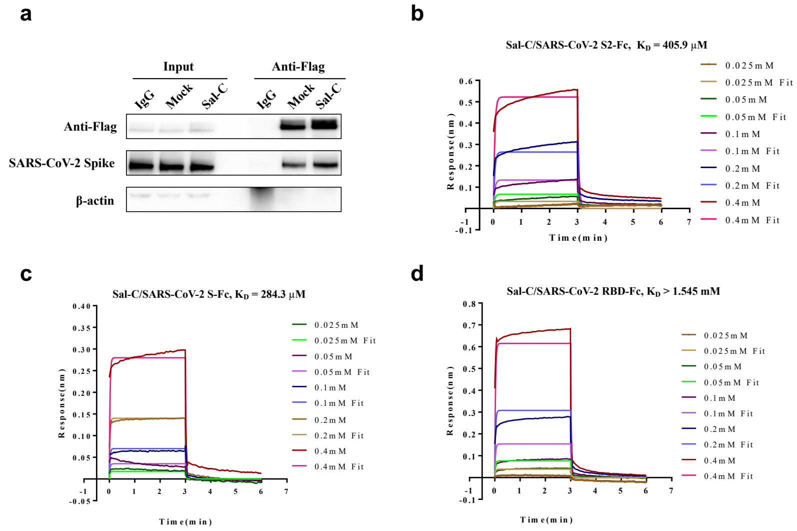

Fig. S3 Sal-C interacts with SARS-CoV-2 spike protein S2 subunit. a Evaluation of Effect of Sal-C on SARS-CoV-2 S protein binding to human ACE2 using HEK293T cells with over-expressed SARS-CoV-2 S and human-ACE2. The binding of SARS-CoV-2 S protein and ACE2 (Anti-Flag) in the presence or absence of Sal-C (5 μM) were detected by Co-IP assays. IgG was included as a negative control. **b-d** Biotinylated SARS-CoV-2 S2-Fc (**b**), SARS-CoV-2 S-Fc (**c**) or SARS-CoV-2 RBD-Fc (**d**) were immobilized on streptavidin (SA) sensors and binding to the gradient concentration of Sal-C was measured with Biolayer Interferometry (BLI) technology on Fortebio octet K2 system.

Table. S1 Binding affinities of Sal-C to the SARS-CoV-2 S2-Fc, SARS-CoV-2 S-Fc and SARS-CoV-2 RBD-Fc, related to Supplementary Fig. S3b-d.

**Sal-C**	**K_D (μM)**	**K_{on} ($M^{-1}s^{-1}$)**	**K_{off} (s^{-1})**	**R^2**
**SARS-CoV-2 S2-Fc**	405.9	1.55E+04	6.27E+00	0.9990
**SARS-CoV-2 S-Fc**	284.3	1.45E+05	4.12E+01	0.9724
**SARS-CoV-2 RBD-Fc**	>1545.0	<3.51E+04	5.42E+01	0.9954

Materials and Methods

Cell lines and Plasmids

HEK293T, 293T/ACE2, 293F cells and Vero-E6 cell lines (ATCC, USA) were cultured in Dulbecco's Modified Eagle Medium (DMEM, Gibco, USA) supplemented with 10% fetal bovine serum (FBS, Capricorn scientific, Germany) and 1% penicillin (100 units/ml) /streptomycin (100 μg/ml) (Gibco, USA). 293T cells stably expressing human-ACE2 (293T/ACE2) were constructed by our laboratory.

The envelope-expressing plasmids of SARS-CoV-2 S (pcDNA3.1-SARS-CoV-2 S and pAAV-IRES-EGFP-SARS-CoV-2 S) were kindly provided by Dr. Lu Lu (Fudan University, China). Plasmid pAAV-IRES-EGFP was purchased from Hedgehogbio Science and Technology Ltd. The luciferase reporter vector (pNL4-3.Luc.R-E-) was maintained in our laboratory. Expression plasmids for full-length vesicular stomatitis virus (VSV) glycoprotein (VSV-G) was obtained from Addgene (Cambridge, MA).

Chemicals, Peptides and Proteins

A collection of 491 natural compound library were purchased from TargetMol (China). Salvianolic acid C (HPLC purity > 99%) was purchased from TargetMol (China).

SARS-CoV-2 HR1P (ANQFNSAIGKIQDSLSSTASALGKLQDVVNQNAQALNTLVKQ) and SARS-CoV-2 HR2P (DISGINASVVNIQKEIDRLNEVAKNLNESLIDLQEL) were synthesized by GL Biochem Ltd.

Biotinylated SARS-CoV-2 S-Fc, SARS-CoV-2 S2-Fc and SARS-CoV-2 RBD-Fc were expressed and purified by our laboratory.

Cell-cell fusion assays

HEK293T cells were transfected with pAAV-IRES-GFP-SARS-CoV-2 S or vehicle pAAV-IRES-GFP to prepare effector cells by using PolyJet (SignaGen, USA). Targeted cells (Vero-E6) were seeded in 96-well plates 6 hours prior to SARS-CoV-2 S-mediated fusion assay. 293T/SARS-CoV-2 S/EGFP or 293T/EGFP effector cells in the presence or absence of Sal-C were overlaid on targeted cells. After 24 hours, three fields were selected in each well randomly and the fused cells were counted under inverted fluorescence microscope (Zeiss, Germany).

Pseudotyped SARS-CoV-2 infection assays

Pseudovirus (PsV) were produced by co-transfection HEK293T cells with pNL4-3.Luc.R-E- and plasmids encoding either SARS-CoV-2 S, or VSV-G by using PolyJet (SignaGen, USA). The supernatant containing PsV were pre-mixed with gradient concentrations of Sal-C for 30 minutes. The mixture were transferred to the 293T/ACE2 or Vero-E6 cells and incubated for 48 hours (triplicate samples). Cells were lysed with 1X culture lysis buffer (Promega, USA) for 15 minutes, and luciferase activity were quantified by measuring luminescence upon addition of luciferase assay substrate (Promega, USA) using a Synergy HTX (Bio Tek, USA).

Authentic SARS-CoV-2 inhibition assay

Authentic SARS-CoV-2 inhibition assay in a full-time model was performed as previously described.[2] Briefly, authentic SARS-CoV-2 (isolate Wuhan-Hu-1) was preserved at Wuhan institute of virology, Chinese Academy of Sciences. Vero-E6 cells were pre-treated with gradient-diluted Sal-C for 1 hour, followed by incubation with SARS-CoV-2 (MOI=0.05) for 1 hour at 37 °C. After that, fresh medium with corresponding-concentration of Sal-C were added and viral RNA were extracted by Viral RNA/DNA Extraction Kit (Takara, Japan) 24 hours later, and viral copies were quantified on ABI 7500 (Takara TB Green® Premix Ex Taq™ II, Japan) with a pair of primers targeting S gene. The forward primer (5'-3'): GCTCCCTCTCATCAG TTCCA; the reverse primer (5'-3'): CTCAAGTGTCTGTGGATCACG.

Indirect immunofluorescence assay

Vero-E6 cells in 48-well plate after authentic SARS-CoV-2 inhibition assay were fixed with 4% paraformaldehyde overnight (Bio-Rad), then permeabilized with 0.2% Triton X-100 (Sigma, USA), followed by immunofluorescence staining of rabbit anti-NP polyclonal antibodies at 4 °C overnight. Goat anti-rabbit IgG H&L (Alexa Fluor® 488) (Abcam) were used as the second antibody. Nuclei were stained with DAPI. Fluorescence images were acquired using Axio Observer microscope (Zeiss, Germany).

Plaque reduction assay

Plaque reduction assay in an Ongoing-infection model or Post-infection model was performed as previously described.[3] Vero-E6 cells were seeded in 24-well plates overnight. For the Ongoing-infection model, cells were firstly pretreated with 50, 16.6 and 5.5 μM Sal-C for

1 hour, then 100 plaque-forming units (PFU) authentic SARS-CoV-2 were added and incubated for 1 hour at 37 °C. For the Post-infection model, cells were firstly incubated with authentic SARS-CoV-2 (100 PFU) at 37 °C for 1 hour, and then, 50, 16.6 and 5.5 μM Sal-C were added to the surface of monolayer cells. Four days later, cells were fixed by 4% paraformaldehyde overnight (Bio-Rad), followed by staining with 1% crystal violet.

Circular Dichroism (CD) Spectroscopy

CD spectra were recorded on a Chirascan plus ACD (Applied Photophysics Ltd, England). HR1P and HR2P were dissolved in buffer (0.1M KCl, 0.05M PO_4, pH 7.2) at a final concentration of 10 μM. Briefly, HR1P were incubated with PBS or Sal-C (10 or 20 μM) at 25 °C for 30 minutes, followed by addition of HR2P (10 μM). After further incubation at 25 °C for 30 minutes, the CD wave scans were measured from 190 to 260 nm at 4 °C with the bandwidth of 2 nm and the step size of 1nm.

Native polyacrylamide gel electrophoresis (N-PAGE)

HR1P and HR2P were dissolved in phosphate buffer (pH 7.4) at a final concentration of 25 μM. HR1P with or without indicated-concentration Sal-C were incubated at 25 °C for 30 minutes, followed by the addition of HR2P. The mixture were incubated for another 30 minutes and segregated by 18% Tris-glycine gel with constant 125V at room temperature for 2 hours. The gel was stained with coomassie blue staining (HaoMa Biotechnology, China) and imaged with a Tanon 2500-B scanner (Tanon Science & Technology).

Molecular Docking

Post-fusion core of 6-HB was selected as the docking structure (pdb code: 6LXT). In this structure, one of the HR2 residues from 1163 to 1197 were removed and the channel occupied by this HR2 was defined as the docking grid, ligands were prepared in MGLTools1.5.4 and docking was performed via auto-dock vina. For each ligand docking, ten binding modes were analyzed and the top one was selected to further manually checking according to the calculated binding affinity.

Western blot (WB)

The S glycoprotein of SARS-CoV-2 in HEK293T cells were detected by using western

blot. Briefly, cells transfected with plasmids encoding S protein of SARS-CoV-2 or VSV-G were lysed at 48 hours post-transfection. Primary anti-SARS S mAb (1:2000) (Sinobiological Inc, China) were incubated overnight at 4°C, followed by secondary horseradish peroxidase (HRP) conjugated antibodies (1:5000) and visualized with Fluor Chem E device (ProteinSimple, USA).

Coimmunoprecipitation (Co-IP)

For coimmunoprecipitation assays, 293T cells transfected with pcDNA3.1-SARS-CoV-2 S (2μg) and pcDH-ACE2-Flag (2μg) by using PolyJet (SignaGen, USA). After incubation at 37°C for 6h, replace fresh DMEM with Sal-C (5μM). After incubation at 37°C for 48 hours, cells were harvested and lysed in IP lysis buffer (Beyotime, China) with protease inhibitor cocktails. Immunocomplexes were solubilized in SDS (5×) loading buffer and immunoblotted with the indicated antibodies by using WB analysis. The input control contained 5% protein.

Biolayer Interferometry (BLI)

The SARS-CoV-2 S-Fc, SARS-CoV-2 S2-Fc or SARS-CoV-2 RBD-Fc proteins expressed and purified from 293F cells were immobilized on the streptavidin (SA) biosensors by pre-labeled biotin with NHS-PEG12-Biotin (Thermo Fisher, USA). Biotin-S-Fc, Biotin-S2-Fc and Biotin-RBD-Fc were diluted in PBS (pH=7.5) to a concentration of 20 μg/ml and loaded for 300 seconds. BLI assays were performed on the FortBio Octet K2 instrument and the affinity constant (K_D), association rate constant (K_{on}) and dissociation rate constant (K_{off}) were calculated using 1:1 binding model on GraphPad Prism software version 8.0.

Statistical analysis

Significance analysis was performed by GraphPad Prism software version 8.0. Differences of inhibition percent, EC_{50} and IC_{50} values were calculated using a one-way analysis of variance (ANOVA). $p < 0.05$ was considered statistically significant.

Reference

1 Xia, S. *et al.* Inhibition of SARS-CoV-2 (previously 2019-nCoV) infection by a highly potent pan-coronavirus fusion inhibitor targeting its spike protein that harbors a high capacity to mediate membrane fusion. *Cell Res* **30**, 343-355 (2020).

2 Wang, M. *et al.* Remdesivir and chloroquine effectively inhibit the recently emerged novel

coronavirus (2019-nCoV) in vitro. *Cell Res* **30**, 269-271 (2020).

3 Jin, Z. *et al.* Structure of M(pro) from SARS-CoV-2 and discovery of its inhibitors. *Nature* (2020).
